# Pandora's Box

**DOI:** 10.1192/bji.2022.15

**Published:** 2022-08

## The magic of our genome

Have you ever wondered how our bodies manage to tightly pack our DNA into the tiny 46 spaces of our chromosomes, bearing in mind that every time a cell divides it creates a 4 m length of DNA. A recent study from EMBL Heidelberg and the Julius–Maximilians Universität Wurzburg aiming to unravel the process examined a family of DNA motor proteins, protein complexes involved in the structural maintenance of chromosomes (SMC). They specifically studied the condensin SMC protein complex, which is essential to the formation of chromosomes. They demonstrated that condensin creates loops of DNA, which may explain how chromosomes are formed.

In order to examine how this works, they designed a number of experiments, which involved observing and manipulating condensin molecules during the process of DNA loop formation. They demonstrated how different parts of this complex act collectively as a molecular machine, with one part anchoring the DNA to hold it steady, while another part acts as a motor that pushes the DNA forward, creating a wide loop. Incredibly, condensin requires minimal cellular energy in the form of adenosine triphosphate for these actions. The authors point out the advances in cryo-electron microscopy that allowed the visualisation of this complex mechanism. This finding opens up a whole new field of study that in the future may be able to look into the control of genes being switched on and off in between cell divisions, improving our understanding of these processes and hopefully even offering the potential for therapeutic interventions in genetic abnormalities.

Shaltiel IA, Datta S, Lecomte L, Hassler M, Kschonsak M, Bravo S,  A hold-and-feed mechanism drives directional DNA loop extrusion by condensin. Science 2022; 376(6597): 1087.3565346910.1126/science.abm4012

## Get your health perceptions right to the benefit of all

In the current climate of global economic turbulence, health economics are of vital importance to the individual, society and the state. Health misperceptions can have serious consequences on health and healthcare utilisation.

In a European study of a population over the age of 50, researchers examined the relationship between health perception and doctor visits, conceptualising health misperception as a result of either overconfidence (measured as overestimation of health) or underconfidence (measured as underestimation of health). Using a survey of health ageing at retirement, they compared objective performance measures and self-reported equivalents to examine the effects of such attitudes. They found that individuals who overestimated their health visited a doctor 17% less often compared with individuals who correctly assessed their health. Individuals who underestimated their health visited a doctor 21.4% more often than those who correctly assessed their health.

These findings have major implications in terms of preventive care and overall health and social care costs.

Spitzer S, Shaikh M. Health misperception and healthcare utilisation among older Europeans. J Econ Ageing 2022; 22:100383.

## Can we change our ‘dark side’?

Whereas in the past people with severe personality disorders were considered untreatable, in recent years there has been increasing interest in attempting to modify some behaviours by psychological means. A recent study sheds light into the possibility of improvements even in the more serious traits of individuals and offers hope for real change.

The author extended his previous findings, which had shown that people do want to change their ‘big five’ personality traits (extraversion, agreeableness, openness, conscientiousness and neuroticism). In this study, he specifically examined the extent to which people want to change what is considered their ‘dark side’, the triad of Machiavellianism, narcissism and psychopathy. In a 16-wave study of weekly interventions, with an intensive longitudinal design, involving 467 participants, he tested whether a desire to change the ‘dark triad’ predicted actual change over a period of 4 months. The participants were also offered an intervention designed to change their ‘big 5’ personality traits, and he tested whether this could also facilitate change in the ‘dark triad’.

Unsurprisingly perhaps, he found that participants in general did not want to change their levels of ‘dark triad’ traits. However, individual variance in the desire to change did predict actual changes in the ‘dark triad’ during the 4 months of the study. He also found that interventions targeting agreeableness enhanced changes in the three ‘dark triad’ traits. So, want it or not, taking steps towards becoming more agreeable may actually make one less Machiavellian, narcissistic and psychopathic. These findings offer some hope for more effective therapeutic treatments for some serious personality disorders. We could we also speculate that incorporating such interventions in approaches to children exhibiting worrying behaviours could possibly reduce the risk of them developing a severe personality disorder in adulthood.

Hudson NW. Lighten the darkness: personality interventions targeting agreeableness also reduce participants' levels of the dark triad. J Pers [Epub ahead of print] 14 Mar 2022. Available from: 10.1111/jopy.12714.35285028

## Our brain betraying our politics?

So far, political ideology and its underpinnings have been investigated using psychological and socioeconomic research approaches. But do our brains have a biological role in determining whether we are liberal or conservative in our political ideology? Researchers from Ohio University say yes!

By means of artificial intelligence methods and convolutional neural networks, they developed predictive models of ideology. They also used functional connectivity from functional magnetic resonance imaging (fMRI) scans for nine standard task-based settings, which they applied using a whole-brain approach to identify which areas of the brain are activated simultaneously, i.e. communicate with each other when performing particular tasks. Data from the Ohio State University Wellbeing project, involving 174 healthy adults performing standard scientific experiment tests while undergoing fMRI scanning, were obtained. Of the eight tasks, three stood out in terms of particular links to ideology. These were (a) the empathy task, where participants were shown photos of neutral, happy, sad and fearful faces; (b) the episodic memory task; and (c) a reward task where participants could lose or win money, dependent on their speed in pressing a button.

The scans of participants performing the reward task predicted political extremism, either very conservative or very liberal, whereas the results for the empathy task were associated with moderate ideology. Activations of the amygdala, inferior frontal gyrus and hippocampus were most strongly associated with political affiliation.

The researchers found that that the brain scans performed as well as parental ideology in predicting political leanings; even more interestingly, when the brain scan results were combined with sociodemographic data (age, gender, income and education), the model was superior to parental ideology for predicting participant ideology.

The authors conclude that although the direction of causality is unclear, the biological and neurological roots of political behaviour may run much deeper than was previously thought.

Yang S-E, Wilson JD, Lu Z-L, Cranmer S. Functional connectivity signatures of political ideology. PNAS Nexus [Epub ahead of print] 23 May 2022. Available from: 10.1093/pnasnexus/pgac066.PMC929124235860601

## From meta-analysis to meta-matching

We know that one hat doesn't fit all, and personalised medicine has become the ideal approach to the management of our patients. But this is not as easy as we would hope. To achieve improved results, we need to consider the symptoms as well as the biology of the individual, refine the diagnosis and treatment, and predict clinical outcomes. At present, the relationship between brain data and clinical outcomes in terms of effect size is low, owing to the lack of large-enough samples.

A recent study proposes an interesting alternative way of assessing data and improving predictive ability in small data-sets. This method, which the authors termed meta-matching, involves small studies ‘piggybacking’ on to sufficiently large data-sets, for example, the UK Biobank and the Adolescent Brain Cognitive Development neuroimaging data-sets.

The method involves the algorithm trained on these large databases being tweaked to predict a slightly different phenotype in a separate smaller data-set. The results showed impressive improvements in the predictive performance of such meta-matching, with a doubling of the accuracy when piggybacking compared with when using the small data-set alone. The authors suggest that this new concept of meta-learning and transfer learning, which has been in used in the field of machine learning, could be applied to human neuroscience. The better matching approach, used on a wide range of phenotypes, including psychiatric diagnosis, treatment outcomes, personality factors or intelligence, may enable more effective personalised medicine.

Bijsterbosch J. Piggybacking on big data. Nat Neurosci 2022; 25: 682–3.3557813310.1038/s41593-022-01058-wPMC9179090

